# Lessons from the COVID-19 pandemic and recent developments on the communication of clinical trials, publishing practices, and research integrity: in conversation with Dr. David Moher

**DOI:** 10.1186/s13063-022-06624-y

**Published:** 2022-08-17

**Authors:** Daeria O. Lawson, Michael K. Wang, Kevin Kim, Rachel Eikelboom, Myanca Rodrigues, Daniela Trapsa, Lehana Thabane, David Moher

**Affiliations:** 1 Department of Health Research Methods, Evidence, and Impact, McMaster University, Hamilton, ON Canada; 2 Department of Medicine, McMaster University, Hamilton, ON Canada; 3 Population Health Research Institute, Hamilton Health Sciences, Hamilton, ON Canada; 4 Biostatistics Unit, Father Sean O’Sullivan Research Centre, St. Joseph’s Healthcare Hamilton, Hamilton, ON Canada; 5 Centre for Journalology, Clinical Epidemiology Program, Ottawa Hospital Research Institute, Ottawa, ON Canada; 6 School of Epidemiology and Public Health, Faculty of Medicine, University of Ottawa, Ottawa, ON Canada

**Keywords:** Clinical trials, Collaborative research, Education, Open science, Publishing, Reporting guidelines, Research integrity

## Abstract

**Background:**

The torrent of research during the coronavirus (COVID-19) pandemic has exposed the persistent challenges with reporting trials, open science practices, and scholarship in academia. These real-world examples provide unique learning opportunities for research methodologists and clinical epidemiologists-in-training. Dr. David Moher, a recognized expert on the science of research reporting and one of the founders of the Consolidated Standards of Reporting Trials (CONSORT) statement, was a guest speaker for the 2021 Hooker Distinguished Visiting Professor Lecture series at McMaster University and shared his insights about these issues.

**Main text:**

This paper covers a discussion on the influence of reporting guidelines on trials and issues with the use of CONSORT as a measure of quality. Dr. Moher also addresses how the overwhelming body of COVID-19 research reflects the “publish or perish” paradigm in academia and why improvement in the reporting of trials requires policy initiatives from research institutions and funding agencies. We also discuss the rise of publication bias and other questionable reporting practices. To combat this, Dr. Moher believes open science and training initiatives led by institutions can foster research integrity, including the trustworthiness of researchers, institutions, and journals, as well as counter threats posed by predatory journals. He highlights how metrics like journal impact factor and quantity of publications also harm research integrity. Dr. Moher also discussed the importance of meta-science, the study of how research is carried out, which can help to evaluate audit and feedback systems and their effect on open science practices.

**Conclusion:**

Dr. Moher advocates for policy to further improve the reporting of trials and health research. The COVID-19 pandemic has exposed how a lack of open science practices and flawed systems incentivizing researchers to publish can harm research integrity. There is a need for a culture shift in assessing careers and “productivity” in academia, and this requires collaborative top-down and bottom-up approaches.

**Supplementary Information:**

The online version contains supplementary material available at 10.1186/s13063-022-06624-y.

## Background

The coronavirus disease 2019 (COVID-19) pandemic has introduced unique challenges and learning opportunities for research methodologists and clinical epidemiologists-in-training. As part of a McMaster University graduate course, experts are invited to share their insights about the current challenges, recent innovations, and future directions in trial methodology. The 2021 Hooker Distinguished Visiting Professor Lecture series featured Dr. David Moher (see Additional file 1), a Professor in the School of Epidemiology and Public Health at the University of Ottawa, where he holds a University Research Chair. Dr. Moher is also the Director of the Centre for Journalology and a Senior Scientist in the Clinical Epidemiology Program at the Ottawa Hospital Research Institute. Dr. Moher received his PhD in Clinical Epidemiology and Biostatistics from the University of Amsterdam. He is one of the founders of the Consolidated Standards of Reporting Trials (CONSORT) statement [1], an internationally and widely endorsed reporting guideline for randomized controlled trials. He also leads the Canadian Enhancing the QUAlity and Transparency Of health Research (EQUATOR) Centre, an international network focused on improving the quality and reporting of health research [2]. Dr. Moher is a world-renowned expert in the science of research reporting having been involved in and leading many other reporting guideline initiatives. He leads a research program investigating predatory journals and current incentives for publishing in academia and works to educate researchers and the next generation of health scientists.

In light of his expertise, we look back on developments in the research community a year since the World Health Organization declared COVID-19 a pandemic [3]. This paper is based on his invited lectures and the student rounds held on March 22, 2021, tailored to the written format. The following sections outline our discussion with Dr. Moher including (1) reporting of trials; (2) research integrity and open science; publishing practices focused on (3) journals, editors, and peer reviewers, and (4) predatory journals; (5) meta-science; (6) research collaboration; and (7) researcher assessment and career advice.

## Main text

### Reporting of trials

Reporting guidelines assist authors with reporting the minimum set of items for a specific type of research. Examples of types of poor reporting in health research include non-reporting or delayed reporting (e.g., published only as a conference abstract), selective reporting (e.g., of primary outcomes, analyses), incomplete reporting (i.e., omitting crucial methods details, missing results and data that cannot be pooled in meta-analyses), spin and misleading interpretations, and inconsistencies between sources (e.g., registration and final report). The publication of the first version of the CONSORT statement in 1996 had major downstream impacts for trial reporting practices. On a broader scale, it has influenced the development and implementation of evidence-based reporting guidelines [4, 5]. Owing to its widespread acceptance, CONSORT has become a part of the “lexicon of trials” says Dr. Moher. He reflects on how this work has influenced similar movements for transparent reporting in other fields including evolutionary biology and ecology [6] and psychology [7, 8] for example. It has led to the development of accompanying resources [9] and advanced publishing practices of prestigious journals which have created dedicated methods and reporting sections in response. Similarly, editorial groups, like the International Committee of Medical Journal Editors (ICMJE) [10] and the World Association of Medical Editors, are more aware of the importance of reporting guidelines.

However, despite the many advances spurred by the development of CONSORT, challenges in communicating trials persist. Dr. Moher discusses how the application and misuse of reporting guidelines has remained problematic and that CONSORT has been used as a “quality control measure” (e.g., in studies evaluating reporting trends over time). He says that this was never its intention, and the developers have explicitly stated that CONSORT should not be used as a scoring system. When asked about how we can better support the ultimate goal of improving the design and reporting of trials (which are often assumed to coincide), Dr. Moher reflects on his discussions with friend and colleague Prof Douglas Altman, “We spoke about this incessantly, the history of CONSORT really is primarily about trying to get people to improve the quality of reporting. It’s possible that if we were able to achieve that, that we might move to work on trying to improve the design of studies. It seems to me that the design of trials is not necessarily a low hanging fruit” as there is rarely one singular or agreed upon approach to the design of trials. He uses the example of randomization methods in COVID vaccine trials as an example, “Interestingly enough, with all the vaccine trials they only used a 1:1 [randomization] ratio, why didn’t they use 2:1? Did they have clinical equipoise, or did they think that the vaccine would be better?” In contrast, “there is only one way to report something, and that is clearly and transparently” irrespective of the study design. He discusses the “excitement” surrounding CONSORT and other similar initiatives as tools that improved the quality of reporting, “These interventions have helped a little ... the evidence is that there are still about 50% of reports of randomized trials that tell you nothing about the randomization process,” a less exciting reality, “I think we still have a way to go.”

As part of the overwhelming volume of COVID-related research [11], many smaller and low-quality studies have been published, along with rapid peer-review and rapid evidence syntheses of these studies. Dr. Moher highlights the paper by London and Kimmelman in which the authors discuss “pandemic research exceptionalism” and how the pandemic has paved the way for questionable research practices at the expense of rigorous, high-quality research [12], which ultimately affects patients and health systems [12]. He also cites work by Goldacre et al. highlighting selective reporting in 58 of 67 trials published during a 6-week period in prominent journals that endorse CONSORT [13]. The authors found that over 300 new, non-prespecified outcomes were silently added to the final trial reports [13]. In another study based on 262 trials reported in the most prominent oncology journals, only 11% reported all 10 essential elements about the intervention (e.g., drug name, dose, route) [14], “Authors cannot adequately describe basic essential information for readers”. Clearly, he says, our current standards for reporting have failed. In terms of how to prevent these issues including the misuse of CONSORT, he says the “reporting of trials is unlikely to change reporting practices unless we invoked policy to change” and that this was an important moment of realization. Specifically, policy mandates at the funder- and institution-level. Funders could require grantees to report their trials using CONSORT. Similarly, universities and other research organizations could require clinical (and pre-clinical) trials conducted at their institutions to use CONSORT when reporting their results. Additionally, the use of reporting guidelines could be incorporated as part of the researcher assessment. For example, when a researcher is providing her curriculum vitae to an assessment committee, each preprint and peer reviewed clinical trial publication could state whether CONSORT was used.

### Research integrity and open science

Research integrity is defined as encompassing honest and verifiable methods, reporting in adherence with rules, regulations, and guidelines, and following commonly accepted professional codes or norms [15], which aligns with the concept of open science [16]. The basis of open science is transparent and accessible knowledge that is shared and developed through collaborative networks [17]. The “open” aspect refers to many different components including openness in data, infrastructure, education, and peer review. Dr. Moher believes that open science and data sharing foster research integrity, promote transparency, are endorsed by patients and are likely catalysts for promoting equity, diversity, and inclusion as the “great equalizer to access”. He says that open science is becoming ubiquitous in Europe and may reduce research waste [18]. He also advises that what we read may depend on the openness and transparency of the journal and not necessarily the authors, and practices such as open peer review promote greater transparency.

In terms of practicing transparency, Dr. Moher believes that when it comes to addressing the issues with reporting (and publishing in general), “we don’t seem to have a research ecosystem that promotes and values audit-and-feedback.” Audit and feedback (A&F) is a practice that motivates behavior change by generating an awareness of current practices and has been observed to perform best when compliance is low in healthcare settings [19]. Dr. Moher brings up the long and positive history of A&F [19, 20]. He is troubled by the fact that neither institutions nor funding agencies collect data to answer questions about open access and reporting practices. He reflects on the announcement of a new mandate by the Wellcome Trust—a global funder for health research—at the beginning of the pandemic, which highlighted the importance of data sharing and encouraged making all research open access [21]. Although this mandate was signed by hundreds of organizations including funding agencies, “few researchers have lived up to this aspiration” he says. Based on an analysis of the first 535 COVID-19 research reports on preprint servers, researchers found that only 21% included data availability statements and 11% made data available in external repositories [22]. This raises concerns about research integrity, which relies on transparency. Dr. Moher says that these findings reinforce the fact that “it doesn’t take much to sign something” and a lack of A&F by organizations and funders obscures the actual failure in research practices. Sadly, he says, “COVID... has shone a very bright light on the very destructive forces of the currency of publication, at any cost”. In line with these concerns, “Retraction Watch in 1 year has noted 89 retractions of COVID-19 research” which does not include papers with an ‘interest of concern’ or other questionable practices, he elaborates. The shift to “rapid” research has also led to these retracted and predatory journal articles being included in systematic reviews.

Even more resounding is the overwhelming support by patients who want their data shared [23, 24]. Recognizing that trials would not happen without patients, Dr. Moher suggests that current practices seldom align with what patients want. Citing ICMJE, “In return for the altruism and trust that make clinical research possible, the research enterprise has an obligation to conduct research ethically and to report it honestly” [25]. For example, some COVID-19 vaccine trials have explicitly refused to grant access to their data, which makes this research impossible to build upon and reproduce.

What will it take to advance the state of the clinical research landscape? “I had sort of an epiphany, of spending much of my career looking at risk of bias and looking at the same result the whole time, either ‘unclear’ or ‘bad’... the epiphany is thinking more about policy and open science.” Dr. Moher suggests that this can be accomplished with a “digital dashboard for researchers, institutions and funders” at a departmental or institutional level, by different clinical fields within an academic hospital, and so on. The dashboard would track the number and quality of trials an investigator has published. As another idea, metrics such as the number of randomized controlled trials for which CONSORT or the Standard Protocol Items: Recommendations for Interventional Trials (SPIRIT) [26] were used to report the trial protocol and final report could be tracked. Such measures, he believes, are essential to promote change at institutions. Based on consultations with organizations and stakeholders, Dr. Moher says that A&F makes sense to institutions and policy makers, “How can we change if we do not know how we are doing?” As an example of what A&F might look like in this context, Dr. Moher presents a graphical comparison of German medical schools based on their trial registrations between 2009 and 2014 [27]. In this sample, a median of 40% of schools had published their trial results. He points out that these findings are not unique to Germany, with Canada likely in line with these findings based on an ongoing analysis [28]. This translates to waste in research, undermines the significant patient and financial investments in trials, and represents “incredibly bad research integrity, incredibly bad scientific integrity” he says. “Without audit and feedback, we’re simply not going to do better.”

On the topic of concerns with transparency, Dr. Moher points out that publication bias is on the rise and that institutions contribute to this issue. Similarly, the prevalence of spin (i.e., distortion of trial results) and selective outcome reporting bias is very high. To address these biases, Dr. Moher says that we can encourage the use of existing tools such as preprints (most journals allowing these), which is an open science practice. This ensures that the original trial results are available and enhances transparency. Preprint use has been more common in some research fields, such as physics, and he believes that institutions should make this practice mandatory for all researchers “in recognition that peer review might change the manuscript by the time it is accepted.” These types of early research reports have had a big impact on human health and risk communication during the pandemic, but to those without professional knowledge, may be considered the same as final, peer-reviewed reports. Preprint results need cautious interpretation until there is sufficient evidence to make more definitive judgments. Most preprint servers indicate on the cover page and every subsequent page that the paper has not been peer reviewed. However, emerging evidence comparing preprints to published (and peer reviewed) reports suggests little difference in terms of results and linguistics.

For researchers concerned with misuse of shared data, he emphasizes that there is currently very little evidence that this leads to any “scoops of intellectual content”. However, there are currently insufficient resources to help academics with data sharing, and he says that this must come from funders and institutions. For example, “data champions” in universities in the UK (e.g., Cambridge) and the Netherlands (e.g., Technical University of Delft) help researchers share their data. Additionally, public funders are needed to support open science research—without which it is difficult to evaluate specific interventions and their impact—and journals and academic institutions need to participate more actively. He recommends the Transparency and Openness Promotion guidelines developed by the Center for Open Science [29]. This has been adapted for institutions and covers standards including study registration, data/materials/code transparency, and so on.

### Publishing practices: journals, editors, and peer reviewers

The responsibility for fostering openness and improvement in the reporting of trials and health research also lies with publishers, journals, and their editors. “COVID has highlighted the very tenuous practice of peer review and editorial practice” says Dr. Moher, “in general, we do not train peer reviewers, a tremendous flaw, nor do we train editors, another flaw”. He provides a clinical analogy, “Do you think you would let me into the OR [operating room] to do surgery without training? Probably not... But why should people review manuscripts without training, why is there a different standard here?” In line with this, Crawley states in a commentary that journal editors have evaded accountability in addressing poor research and publishing misconduct based on the ICMJE recommendations, where “authors are assigned ‘responsibilities’; journal editors are assigned ‘freedoms’” [30].

We discuss some of the challenges with investigating editorial and peer review practices. Publons (now acquired by Clarivate Analytics), an organization and online platform that helps researchers document their peer review activities, offers free training in peer review (now Web of Science Academy). Dr. Moher discusses his hopes to evaluate the training as an intervention, which was met with great interest from publishing houses but little fiscal commitment, describing it as “a sad story.” This experience emphasizes the challenges faced by the research community, particularly in Canada, to obtain funding from entities such as the Canadian Institutes for Health Research (CIHR) for this type of research. Unfortunately, research on research and publishing practices is “low on the totem pole and it’s very sad because it has tremendous impact and consequence.” Elaborating on the lack of evidence on editor and reviewer core competencies [31], “we have editors and peer reviewers, and we don’t know if they’re any good or not.” For example, he describes the WebCONSORT intervention which was evaluated as a writing tool to help authors with reporting during submission. The intervention was found to be ineffective, partly owing to the more than 100 studies that were incorrectly labeled as randomized controlled trials by editorial offices of 46 journals [32], “a shocking result that editorial offices don’t even know a randomized trial when they see one.”

Despite these limitations, Dr. Moher stresses that peer review is very important and encourages young researchers to participate as a way of “paying it back and paying it forward”. In discussing his approach, “I try to make my peer review evidence-based; it is not the opinion of David Moher...here is the facts and here is the evidence.” He mentions the CONSORT-Based Peer-Review Tool (COBPeer) as another good outcome of CONSORT and that it was effective in helping peer reviewers to identify inadequate reporting [33]. But training and participation in peer review should be incentivized. Unfortunately, with traditional researcher assessment incentives, researchers get little meaningful credit for peer review. More basic, is that universities, where a lot of peer review takes place, do not provide any training to be a peer reviewer. This produces a research ecosystem with untrained faculty and staff providing an intervention (i.e., peer review) for which they are not trained. Like many other staff and faculty requirements at universities, a progressive initial step would be to include mandatory peer review training for all incoming graduate students, including medical trainees, staff, and faculty. Such a course and other publication science activities (e.g., roles such as editor, editorial board member and mentors) could be explicitly included as part of the researcher assessment.

### Publishing practices: predatory publishers

Predatory publishers and journals also have a significant impact on the outputs of health research. Based on an international consensus definition, predatory journals are entities that prioritize self-interest at the expense of scholarship and are characterized by false or misleading information, deviation from best editorial and publication practices, a lack of transparency, and/or the use of aggressive and indiscriminate solicitation practices [34]. Many retractions come from predatory journals, and these corrupt the open access model by attracting researchers with lower Author Processing Charges (APCs). “There are many people who don’t think predatory journals are a problem” says Dr. Moher, citing common misconceptions including that these articles are not cited (evidence shows otherwise, [personal communication, David Moher]), or that they only represent small studies, and so on. A bibliometric study identified that 8.5% of articles included in Cochrane reviews were published in predatory journals [35], demonstrating that “predatory journals have leaked into trusted sources.” He discusses another analysis of studies published in predatory journals including those with ethics approval, from prestigious institutions (e.g., Mayo Clinic), and the majority (57%) from high and upper-middle income countries [36]. Unfortunately, there are many examples of legitimate, publicly funded research (e.g., National Institutes of Health and CIHR) that has ended up in predatory journals. The OMICS publishing group, based in India, is the largest predatory publisher with over 700 predatory health journals and over 2000 fake conferences [37]. With respect to COVID-19, one analysis has found that 367 articles were published in predatory journals between January and May 2020 alone, with some of these journals being indexed in PubMed and MEDLINE [38]. Overall, predatory publishers contribute to both the misinformation and loss of valuable epidemiological information [38], and damage the reputation of researchers and institutions.

Trainees and young researchers are often more susceptible to calls from predatory journals due to less experience with publishing. But Dr. Moher clarifies that all researchers, including senior researchers, can get entangled, and directs us to guidance [39] to help avoid such traps. He encourages us to discuss the appropriateness of journals before submission with colleagues, mentors, and other researchers that are knowledgeable about the publishing landscape (e.g., “Has your research team heard of the journal before?”) “The real competitor of a predatory journal is an open-access journal,” he says, and not subscription-based journals apart from the COVID-19 research that has been made available by several prestigious journals. He recommends checking the Directory of Open Access Journals [40] to confirm if the journal is listed, and if not, to not submit. Similarly, the Liège Library, Belgium, has developed a tool—their algorithm methodology is publicly posted—which allows users to evaluate the authenticity of a journal [41]. There is also the ‘Think. Check. Submit’ tool [42]. He says that institutions should provide mandatory training to graduate students, researchers, and librarians on best publishing practices, including how to select a journal. “At my institution, I have to do WHMIS [Workplace Hazardous Materials Information System] training once a year, I don’t even know what a lab looks like, I couldn’t tell you one end of a lab from another, but I’m required... and if I don’t do it, my email is shut off. So why wouldn’t we have something similar for research integrity, isn’t that important? Why wouldn’t we have that as part of the mandate for ensuring that people know about predatory journals and know about a lot of other publication science issues?”

### Meta-science for advancing research integrity

To better evaluate research integrity and gaps in publishing practices, we discuss the study of research itself. Meta-science—also called research-on-research, meta-research, and many other names—has been described as the scientific discipline that seeks to evaluate the practice of research [43]. This field has grown rapidly in recent years in response to concerns with health research conduct and reporting [44, 45]. Is this type of research providing useful contributions towards the effort to improve reporting practices, to attain some ideal state of “optimal” reporting? Dr. Moher believes that meta-science is very important and “a place to invest intellectual energy in”. He highlights several organizations including the Meta-Research Innovation Center at Stanford (METRICS), California, USA, the Research on Research Institute in the UK, and the Quality | Ethics | Open Science | Translation (QUEST), Berlin Institutes of Health, Berlin, Germany. He notes that much of the groundwork and initiatives are being led by Europe, with Canada slow to respond particularly due to lack of funder support. He says that reporting guidelines like CONSORT, SPIRIT, and PRISMA have been influential in this field, “I think meta-science is here to stay, in a very big way,” elaborating that by helping to evaluate reporting across journals and disciplines, this field will play a key role. “I think meta-science is going to be very important when they have built in audit and feedback” he says and that this is illustrated in a real-world example with the Charité Hospital in Berlin, one of the largest teaching hospitals in Europe. A live, automated dashboard pulls metrics and metadata about trial registration, open access publications, and preprints. This information is currently available at the institutional level. Dr. Moher says that this can be easily applied to measure the use of reporting guidelines by researcher, for example, and allows to make useful comparisons within and between different fields of clinical research or between different institutions. He believes that “this will be part of the meta-science revolution that is coming” and that this will help remedy issues by informing policy, and fostering openness in health research conduct, reporting, and data sharing.

In addition to meta-science, Dr. Moher believes that the use of technology will have a big impact on advancing meta-science and reporting guidelines. He gives an example of an artificial intelligence-based CONSORT compliance tool (which is currently being tested by some publishing houses) to evaluate compliance at the manuscript submission stage [46]. “Industry do a far better job than we do on reporting and conducting trials, and we have evidence of that”. However, in academia, he again highlights the importance of metrics related to openness and transparency in research, as opposed to quantity-oriented metrics that focus on journal impact factors and number of publications, “We need to start assessing people’s career on using things like reporting guidelines”. On invoking policies, “Any new faculty, student, or staff... should be required to take a course on research integrity that would include sessions on reporting, because reporting is very much part of this whole notion of trustworthiness and research integrity”. There is a need for both a top-down and bottom-up approach to invoke such policies. What should we, as a collective of researchers and institutions, do next? “I think we need to put all our energies on improving the completeness and transparency of reporting” he says, principally by “getting reporting guidelines used, and getting policies changed in academic institutions to reward people for good scientific behavior”. He says that these ongoing challenges—limited open access research, academic incentives to publish by quantity over quality, lack of training of journal editors and peer reviewers, and predatory publishers— have been exacerbated because of the COVID-19 pandemic, “it is too early to know, in general, what the quality reporting is overall.” He believes that these are the broader, important issues that need to be addressed through meta-science to make explicit conclusions and inform policy. When asked about what a research integrity-oriented policy might look like (e.g., encouraging researchers to include the reporting guideline used for every publication on their curriculum vitae), “I wouldn’t use the language encourage; I would use far stronger language. Encourage is very open to interpretation.”

### Team science and collaborations

Next, we discussed research collaborations, an area in which Dr. Moher has demonstrated incredible talent, having led numerous successful initiatives. Any advice for working with diverse groups of researchers and stakeholders? “Make it about the team, not about the individual,” he attributes any success of initiatives like CONSORT and the Preferred Reporting Items for Systematic Reviews and Meta-Analyses (PRISMA) as a result of the team, “It is a team effort, it’s not an effort of David Moher.” Elaborating on international collaborations and cross-cultural differences, “How the message is delivered is critical” and paying attention to clarity in communication is essential. He points out the importance of working collaboratively, especially in spaces where big personalities can impede these processes, “Never stop listening to collaborators, including the difficult ones, and in academia, there can be an over-representation of difficult ones.” However, he cautions, “Try to avoid including people who find it difficult to play in the sandbox, or compromise,” admitting that he has had to terminate collaborations with disruptive individuals. He encourages us to stay humble and lead by example in what we do, as that is often much more enduring than what we say. “I think it’s really important to empower everybody” on his approach to promoting diverse and collaborative science, “You will definitely hear from the extroverts, but you need to give time to the introverts,” a statement that will likely resonate with many scientists, particularly as many have been thrust into the spotlight during the COVID-19 pandemic. Since the pandemic, the shift in the approach to science communication requires us to also communicate outside of our collegial circles and with multiple audiences. He emphasizes the need to stand by one's principles when pressured to report non-evidence-based information. To help researchers, journals should institute requiring a lay summary as part of any published article. Similarly, we need to augment patient and public involvement and engagement in research which will facilitate scientific communication. For this reason, we should also consider adding patient/public membership to journal editorial boards. 

### Researcher assessment for academic promotion and career advice for young scientists

Traditional publishing practices, as it relates to academic career advancement, are a major culprit in the persistent challenges with reporting of health research. Dr. Moher reflects on merit evaluations by academic institutions which reinforce these practices. Specifically, the “publish or perish” paradigm requiring researchers to report their annual number of publications and the proportion published in journals with a journal impact factor (JIF) greater than x. To what degree do metrics such as JIF matter? “Impact factors tell us nothing about the quality of the research, it’s an erroneous measure to use” he says, it indicates nothing about the authors yet it remains “ubiquitous in assessing researchers.” He believes this is a major flaw and describes his recent work evaluating the criteria for promotion and tenure in faculties of medicine within the U15, a collective of Canada’s self-appointed research-intensive universities. Progressive criteria (labeled as ‘nontraditional’ in the paper) included aspects such as data sharing, and it was found that universities based their assessments on traditional criteria without considering open science practices [47].

Dr. Moher elaborates on the competing forces between researchers and institutions, “I think our energies need to be based on changing policies at academic institutions about how researchers are rewarded.” The wide adoption of JIF by leadership is likely related to its ease of collection, “doesn’t matter whether it makes sense or not, it’s easy to collect.” He notes that JIF is associated with university ranking schemes, which are partly based on “productivity” as measured by numbers of publications, “ranking schemes, systems that are equally as problematic as journal impact factor.” This system also impacts students who are required to publish papers to complete their PhD. He directs us to the Declaration on Research Assessment (DORA) which is largely based on the belief, backed by evidence, that universities should not use JIF to assess researchers [48]. He encourages researchers and institutions to sign DORA and highlights the Hong Kong principles [49] which focus on research integrity. “We have to work hard to change that” with DORA and the Hong Kong principles as some ways to help the research community move towards incentivizing ethical publishing practices and scientific integrity (i.e., is the researcher trustworthy? Not, does the researcher publish a lot?) He says that this requires a culture change, and that these initiatives (e.g., revamping career assessment metrics) have a much larger movement outside of Canada. He says that there is a need for this change to stem from institutions and funders by way of policy. However, a bottom-up approach may be equally important. Researchers may come up against numerous obstacles in tackling these issues from within institutions. He encourages starting grassroots committees as a way of gaining credibility with institutional leadership, pointing to similar initiatives in many universities in Europe [50], “these committees are not very easy for leadership to ignore.” He also suggests looking at progressive institutions like the Leiden University, University of Ghent, Belgium and Utrecht University, Netherlands which have set examples of how this can be accomplished collaboratively. He believes that collaborative efforts must be at the heart of it all; it “reinforces the notion that we have in moving this forward is always to do it with an integrative knowledge translation approach,” which includes asking both researchers and institutions about their values.

We finish the discussion by asking Dr. Moher about his career mistakes and challenges. Have any of these experiences turned out to be the most rewarding? Reflecting on career successes and his “many failures,” he offers insights including personal struggles with dyslexia, “Everybody told me I would never succeed, and the people who really emphasized that I would never succeed were schoolteachers and university professors”. On many “no’s” and negativity, “don’t take it personally even if it’s meant personally,” something that is very prevalent in academia he says, and urges trainees to surround themselves with mentors who will help them, and to stay focused. “In the end of the day it’s about the methodology of what one is trying to do.” This advice aligns closely with that of his late colleague, Prof Doug Altman, who also spoke about the need for getting “good research practices out there into early career training before people develop bad ideas.” [51] There should also be reflection and recognition that research is not for everybody. As we have heard extensively, Dr. Moher says we also need good educators and scientists who can tackle policy.

He also discusses the importance of capacity-building and mentoring the next generation, “I definitely want to empower mentees.” He pays close attention to including younger researchers, minorities, and women researchers, in particular, in his work. Dr. Moher discusses the challenges for this group, citing work by Professor Witteman and colleagues that found policies such as those at CIHR inherently discriminate against female research applicants [52]. “Women researchers have fared very, very badly during COVID-19” he says, with issues relating to gender and equity exacerbated by additional responsibilities that women are expected to take on outside of academia [53]. He concludes with optimism, “I feel the future is in very good hands... Never stop learning, never stop listening, never stop reading.”

## Conclusion

Trust in trials and more broadly, scientific research, has been negatively impacted during the COVID-19 pandemic, partly due to the miscommunication of science. Several approaches by researchers can help to improve scientific integrity and regain public trust in science, including transparent reporting and open science practices such as data sharing. In contrast to the more traditional approaches for incentivizing career progression (i.e., quantitative), Dr. Moher’s outlook and messages concerned with open science, if adopted more broadly, suggest the possibility for a research career that may be more sustainable for students considering a future in academia. Open science practices would positively impact the enterprise of research itself with the focus on more qualitative metrics such as registration of research and use of reporting guidelines. How quickly institutions align with and get up to speed with valuing these practices is uncertain, but there are examples that demonstrate this is possible, with various institutions in Europe leading the way. As students, through a more grassroots approach, we can help to educate and advocate for the importance of these practices, and challenge institutions to look critically at research and individual academic output, beyond the simpler assessment of quantity of one’s publications.

## Supplementary Information

**Additional file 1.** Dr. David Moher curriculum vitae.

## Data Availability

Data sharing is not applicable as no datasets were generated or analyzed for this article.

## Abbreviations

A&F: Audit and feedback
CIHR: Canadian Institutes for Health Research
COBPeer: Consort-Based Peer-Review Tool
CONSORT: Consolidated Standards of Reporting Trials
COVID-19: Coronavirus disease 2019
DORA: Declaration on Research Assessment
EQUATOR: Enhancing the QUAlity and Transparency Of health Research
JIF: Journal impact factor
ICMJE: International Committee of Medical Journal Editors
PRISMA: Preferred Reporting Items for Systematic Reviews and Meta-Analyses
SPIRIT: Standard Protocol Items: Recommendations for Interventional Trials
WHMIS: Workplace Hazardous Materials Information System

## References

[1] Moher D, Hopewell S, Schulz KF, Montori V, Gotzsche PC, Devereaux PJ (2010). CONSORT 2010 explanation and elaboration: updated guidelines for reporting parallel group randomised trials. BMJ..

[2] Enhancing the QUAlity and Transparency Of health Research (EQUATOR) Network Oxford, United Kingdom: University of Oxford; 2021 [Available from: https://www.equator-network.org/]. Accessed 19 Apr 2021.

[3] WHO Director-General's opening remarks at the media briefing on COVID-19 - 11 March 2020. World Health Organization; 2020. Available from: https://www.who.int/director-general/speeches/detail/who-director-general-s-opening-remarks-at-the-media-briefing-on-covid-19---11-march-2020. Accessed 19 Apr 2021.

[4] Begg C, Cho M, Eastwood S, Horton R, Moher D, Olkin I (1996). Improving the quality of reporting of randomized controlled trials. The CONSORT statement. JAMA.

[5] Altman DG, Simera I (2016). A history of the evolution of guidelines for reporting medical research: the long road to the EQUATOR Network. J R Soc Med.

[6] O'Dea RE, Lagisz M, Jennions MD, Koricheva J, Noble DWA, Parker TH (2021). Preferred reporting items for systematic reviews and meta-analyses in ecology and evolutionary biology: a PRISMA extension. Biol Rev Camb Philos Soc.

[7] Publications APA, Communications Board Working Group on Journal Article Reporting S (2008). Reporting standards for research in psychology: why do we need them? What might they be?. Am Psychol.

[8] Appelbaum M, Cooper H, Kline RB, Mayo-Wilson E, Nezu AM, Rao SM (2018). Journal article reporting standards for quantitative research in psychology: The APA Publications and Communications Board task force report. Am Psychol.

[9] Keech A, Gebski V, Pike R (2007). Interpreting and reporting clinical trials. A guide to the CONSORT statement and principles of randomised controlled trials.

[10] Recommendations for the Conduct, Reporting, Editing, and Publication of Scholarly Work in Medical Journals. International Committee of Medical Journal Editors; 2019. Available from: http://www.icmje.org/icmje-recommendations.pdf. Accessed 19 Apr 2021. 25558501

[11] Quinn TJ, Burton JK, Carter B, Cooper N, Dwan K, Field R (2021). Following the science? Comparison of methodological and reporting quality of covid-19 and other research from the first wave of the pandemic. BMC Med.

[12] London AJ, Kimmelman J (2020). Against pandemic research exceptionalism. Science..

[13] Goldacre B, Drysdale H, Dale A, Milosevic I, Slade E, Hartley P (2019). COMPare: a prospective cohort study correcting and monitoring 58 misreported trials in real time. Trials..

[14] Duff JM, Leather H, Walden EO, LaPlant KD, George TJ (2010). Adequacy of published oncology randomized controlled trials to provide therapeutic details needed for clinical application. J Natl Cancer Inst.

[15] What is Research Integrity Bethesda, Maryland. National Institutes of Health; 2018. Available from: https://grants.nih.gov/policy/research_integrity/what-is.htm. Accessed 19 Apr 2021.

[16] Laine H. Open science and codes of conduct on research integrity. Informaatiotutkimus. 2018;37(4):48–74.

[17] Vicente-Saez R, Martinez-Fuentes C (2018). Open Science now: a systematic literature review for an integrated definition. J Bus Res.

[18] Glasziou P, Chalmers I (2018). Research waste is still a scandal—an essay by Paul Glasziou and Iain Chalmers. BMJ..

[19] Ivers N, Jamtvedt G, Flottorp S, Young JM, Odgaard-Jensen J, French SD (2012). Audit and feedback: effects on professional practice and healthcare outcomes. Cochrane Database Syst Rev.

[20] Gawande A (2010). The checklist manifesto : how to get things right.

[21] Carr D (2020). Sharing research data and findings relevant to the novel coronavirus (COVID-19) outbreak: Wellcome Trust.

[22] Sumner J, Haynes L, Nathan S, Hudson-Vitale C, McIntosh LD. Reproducibility and reporting practices in COVID-19 preprint manuscripts. medRxiv. 2020; 2020.03.24.20042796:1–11.

[23] Kim J, Kim H, Bell E, Bath T, Paul P, Pham A (2019). Patient Perspectives about decisions to share medical data and biospecimens for research. JAMA Netw Open.

[24] Mello MM, Lieou V, Goodman SN (2018). Clinical trial participants’ views of the risks and benefits of data sharing. N Engl J Med.

[25] De Angelis C, Drazen JM, Frizelle FA, Haug C, Hoey J, Horton R (2004). Clinical trial registration: a statement from the International Committee of Medical Journal Editors. CMAJ..

[26] Chan AW, Tetzlaff JM, Altman DG, Laupacis A, Gotzsche PC, Krleza-Jeric K (2013). SPIRIT 2013 statement: defining standard protocol items for clinical trials. Ann Intern Med.

[27] Wieschowski S, Riedel N, Wollmann K, Kahrass H, Muller-Ohlraun S, Schurmann C (2019). Result dissemination from clinical trials conducted at German university medical centers was delayed and incomplete. J Clin Epidemiol.

[28] Alayche M, Cobey KD, Masalkhi M, Willis JV, Ng JY, Chan A-W (2021). Cross-sectional study evaluating the prevalence of publication bias in trials conducted in Canada.

[29] Nosek BA, Alter G, Banks GC, Borsboom D, Bowman SD, Breckler SJ (2015). Promoting an open research culture. Science..

[30] Munafo MR, Hollands GJ, Marteau TM (2018). Open science prevents mindless science. BMJ..

[31] Moher D, Galipeau J, Alam S, Barbour V, Bartolomeos K, Baskin P (2017). Core competencies for scientific editors of biomedical journals: consensus statement. BMC Med.

[32] Hopewell S, Boutron I, Altman DG, Barbour G, Moher D, Montori V (2016). Impact of a web-based tool (WebCONSORT) to improve the reporting of randomised trials: results of a randomised controlled trial. BMC Med.

[33] Chauvin A, Ravaud P, Moher D, Schriger D, Hopewell S, Shanahan D (2019). Accuracy in detecting inadequate research reporting by early career peer reviewers using an online CONSORT-based peer-review tool (COBPeer) versus the usual peer-review process: a cross-sectional diagnostic study. BMC Med.

[34] Grudniewicz A, Moher D, Cobey KD, Bryson GL, Cukier S, Allen K (2019). Predatory journals: no definition, no defence. Nature..

[35] Hayden JA (2020). Predatory publishing dilutes and distorts evidence in systematic reviews. J Clin Epidemiol.

[36] Moher D, Shamseer L, Cobey KD, Lalu MM, Galipeau J, Avey MT (2017). Stop this waste of people, animals and money. Nature..

[37] OMICS International Hyderabad, India: OMICS International. Available from: https://www.omicsonline.org. Accessed 19 Apr 2021.

[38] Vervoort D, Ma X, Shrime MG (2020). Money down the drain: predatory publishing in the COVID-19 era. Can J Public Health.

[39] Lalu MM, Shamseer L, Cobey KD, Moher D (2017). How stakeholders can respond to the rise of predatory journals. Nat Hum Behav.

[40] The Directory of Open Access Journals. Available from: https://doaj.org. Accessed 19 Apr 2021.

[41] Compass to Publish Liège, Belgium: ULiège Library. Available from: https://app.lib.uliege.be/compass-to-publish/. Accessed 19 Apr 2021.

[42] Think. Check. Submit. 2021. Available from: https://thinkchecksubmit.org. Accessed 19 Apr 2021.

[43] Ioannidis JP, Fanelli D, Dunne DD, Goodman SN (2015). Meta-research: evaluation and improvement of research methods and practices. PLoS Biol.

[44] Ioannidis JP, Greenland S, Hlatky MA, Khoury MJ, Macleod MR, Moher D (2014). Increasing value and reducing waste in research design, conduct, and analysis. Lancet..

[45] Chalmers I, Glasziou P (2009). Avoidable waste in the production and reporting of research evidence. Lancet..

[46] Houle T, DeVoss C. StatReviewer: Automated Statistical Support for Journals and Authors 2018 [Available from: http://www.statreviewer.com]. Accessed 19 Apr 2021.

[47] Rice DB, Raffoul H, Ioannidis JPA, Moher D (2020). Academic criteria for promotion and tenure in biomedical sciences faculties: cross sectional analysis of international sample of universities. BMJ..

[48] Declaration on Research Assessment (DORA) [Available from: https://sfdora.org]. Accessed 19 Apr 2021.

[49] Moher D, Bouter L, Kleinert S, Glasziou P, Sham MH, Barbour V (2020). The Hong Kong Principles for assessing researchers: Fostering research integrity. PLoS Biol.

[50] Woolston C. University drops impact factor. Staff at Utrecht University will be assessed through committment to open science. Nature. 2021;595.

[51] Interview with Professor Doug Altman: Methods in Research on Research; 2017 [Available from: http://miror-ejd.eu/2018/03/20/interview-with-professor-doug-altman/]. Accessed 19 Apr 2021.

[52] Witteman HO, Hendricks M, Straus S, Tannenbaum C (2019). Are gender gaps due to evaluations of the applicant or the science? A natural experiment at a national funding agency. Lancet..

[53] Kreeger PK, Brock A, Gibbs HC, Grande-Allen KJ, Huang AH, Masters KS (2020). Ten simple rules for women principal investigators during a pandemic. PLoS Comput Biol.

