# Supplementary material for: Lessons from the COVID-19 pandemic and recent developments on the communication of clinical trials, publishing practices, and research integrity: in conversation with Dr. David Moher
Source: Trials. 2022 Aug 17;23:671. doi: 10.1186/s13063-022-06624-y (PMC9383655; doi:10.1186/s13063-022-06624-y)
Supplement: Supplementary file 1 — Additional file 1. Dr. David Moher curriculum vitae. [file 13063_2022_6624_MOESM1_ESM.pdf]

The Ottawa Hospital | L'Hôpital d'Ottawa
Research Institute | Institut de recherche

David Moher, MSc, PhD

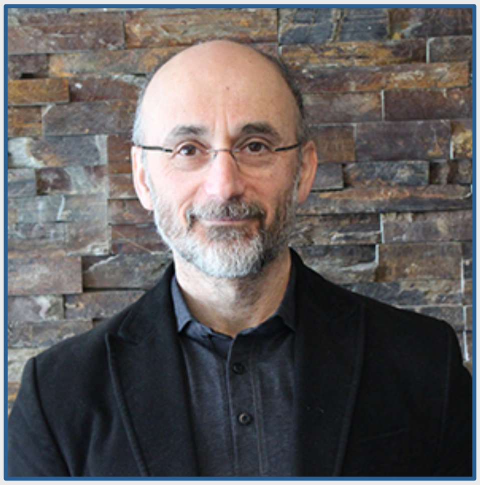

PROFILE

Dr. David Moher is a Professor in the School of Epidemiology and Public Health, University of Ottawa. Dr. Moher established and directs the Centre for Journalology at the Ottawa Hospital Research Institute. His research focus is open scholarship and trustworthiness in research. A world recognized scientific leader in the methodology of systematic reviews and the development of reporting guidelines for health research studies, Dr. Moher has led the development of consensus standards for the reporting of cluster randomized trails (CONSORT), systematic reviews of randomized trials (QUORUM), systematic reviews of observation studies (MOOSE) and diagnostic studies (STARD). Dr. Moher has been a Clarivate Analytics 'Most Cited Researcher in the World' on several occasions, and his research has been cited more than a third of a million times.

CONTACT

http://www.ohri.ca/journalology/

Tel: 1-613-737-8899 ext. 79424
Email: dmoher@ohri.ca
Twitter: @dmoher

HOBBIES

- Reading; Movies; Comedy
- Grandchildren

CURRENT ROLES

- Professor, School of Epidemiology and Public Health, University of Ottawa
- Director, Centre for Journalology, Ottawa Hospital Research Institute
- Senior Scientist, Clinical Epidemiology Program, Ottawa Hospital Research Institute

EDUCATION

- **Ph.D.,** Clinical Epidemiology and Biostatistics, Academic Medical Center, University of Amsterdam, the Netherlands (2004)
- **M.Sc.,** Community Health and Epidemiology, Queen's University, Canada (1986)
- **B.A. (Hons),** Psychology, Queen's University, Canada (1983)

PROFESSIONAL RECOGNITION

- Fellow, Royal Society of Canada (2020)
- Fellow, Canadian Academy of Health Sciences (2019)
- Dr. J. David Grimes Career Achievement Award, Ottawa Hospital Research Institute (2015)
- Clarivate Analytics Highly Cited Researchers in Social Sciences and Clinical Medicine categories, (2017 - 2020)
- One of the most influential researchers in biomedicine (top 0.03% - Boyack KW et al., 2014)
- List of highly influential biomedical researchers (1996 - 2021)
- Bill Silverman Award, Cochrane Collaboration (2009)

(INTER)NATIONAL REPUTATION INDICATORS OR ROLES

- Research Fellow, School of Information Sciences at the University of Illinois at Urbana-Champaign (2020 - present)
- Committee Member, TOP Guidelines, Centre for Open Science (2020 - present)
- Council Member, Ottawa Hospital Research Institute, Equity, Diversity and Inclusion Council (2020 - present)
- Ad Hoc University Committee on Sustainable Library Collections, Committee Member (2019 - present)
- Associate Director, International Congress on Peer Review and Scientific Publication (2018 - present)
- University Research Chair in Journalology, University of Ottawa (2016 - 2021)
- Health Research Board-Trials Methodology Research Network (2016 - present)
- PLOS ONE Human Research Advisory Group (2014 - present)
- Co-Editor-in-Chief, Systematic Reviews Journal (2011 - 2020)

LIFETIME SCHOLARSHIP METRICS

- Peer-reviewed publications: **> 700** | Books/chapters: **11**
- Citations: **> 400,000**
- H-index (Google Scholar): **158**
